# “ZARPAR”—Educational Program for Cognitive and Behavioral Development: Results of an Experiment to Evaluate Its Impact on Antisocial and Pro-Social Behavior

**DOI:** 10.1177/0306624X231172645

**Published:** 2023-05-13

**Authors:** Gilda Santos, Margarida Santos, David P. Farrington, Cândido da Agra, Josefina Castro, Carla S. Cardoso

**Affiliations:** 1 University of Porto, Portugal; 2 University Lusíada, Porto, Portugal; 3 Center for Legal, Economic, and Environmental Studies, Lisboa, Portugal; 4 Interdisciplinary Research Centre on Crime Justice and Security, Porto, Portugal; 5 University of Cambridge, UK

**Keywords:** program evaluation, experimental research, developmental prevention, child skills trainning, antisocial behavior, pro-social behavior

## Abstract

Using an experimental design and a multi-measure and multi-informant approach, the current study sought to evaluate the impact of the early developmental prevention program “ZARPAR”—an intervention designed as a social and cognitive skills training program, that seeks to promote children’s behavioral adjustment. A sample of elementary school children (experimental group *n* = 37; control group *n* = 66), attending Portuguese schools, was assessed before and 6 months after the intervention on the program’s key-dimensions: behavioral problems, social skills, and executive functioning. Based on parent and teacher reports, the results largely suggested that the intervention had no effect or, for some dimensions, even the existence of negative outcomes. Possible reasons for these results are discussed. The current study highlights that, despite the overwhelmingly positive message about developmental prevention programs, not all interventions work, thus reinforcing the need for rigorous evaluations, in order to enhance the success of future interventions.

## Introduction

### The Logic of Risk-Focused Prevention

Since the 1990s, the central notions of the developmental prevention model have become more prominent in intervention research. Increasingly, a large body of academics, decision-makers, and practitioners have reached the conclusion that a remedial attitude toward problematic behaviors is not enough, and have started advocating for the need to intervene early in the life course in order to prevent the development and strengthening of these behaviors (Farrington & Welsh, 2007; Tremblay, 2010).

Simply put, this prevention model, which is nested within the prominent developmental criminology, involves the organized provision of strategies to intervene in individuals, families, schools, or communities in order to prevent the development of problematic behaviors, including antisocial and delinquent ones. The fundamental aim of this model is to identify risk and protective factors for a certain type of disruptive or problematic behavior and to define strategies to reduce the risk factors and to enhance the protective factors across the multiple domains of a child’s functioning (Fagan & Catalano, 2013; Homel, 2005; Reinke et al., 2009). Within this framework, and based on several theoretical models and multiple empirical studies, many risk and protective factors, on different levels, such as the individual (e.g., empathy, executive functioning) family (e.g., parental practices, family structure), school (e.g., school climate, school involvement), or community levels (e.g., social disorganization, collective efficacy) have been associated with these behaviors (Moffitt et al., 2011; Wasserman & Seracini, 2001; Welsh & Farrington, 2012).

Thus, even though the influence of risk and protective factors is probabilistic rather than deterministic, over the past few years, an extensive body of research has shown that children who are exposed to a combination or accumulation of risk factors during different spheres of their lives are at greater risk of developing problematic behaviors in the future. Therefore, based on this rationale, several early developmental prevention programs have been designed. It is well known that these programs might have different types, goals, or formats. Nevertheless, it is generally accepted that it is essential that these interventions should be implemented as early as possible within the child’s life course, during elementary school or, whenever possible, even sooner (Thornberry et al., 1995; Tremblay et al., 2003).

Despite the great diversity of the existing intervention strategies, there are certain types of programs that have been consistently considered promising or effective in enhancing children’s adjustment. At the family level, several studies revealed that different early parenting interventions, mainly focused on *general parent education* and *parent management training*, were effective (e.g., Farrington & Welsh, 2003; Piquero & Jennings, 2012; Welsh & Farrington, 2012). At the contextual level, several intervention strategies, either centered on the peer group (e.g., *peer mediation; peer counseling*), the school (e.g., *reorganization of grades; classroom or institutional management*) or the community (e.g., *after-school programs, community-based mentoring*) were also considered to be effective (e.g., Durlak et al., 2010; S. Wilson & Lipsey, 2007). Finally, at an individual level, there are also intervention strategies considered to be effective in enhancing a child’s social adjustment and in reducing antisocial behaviors, namely those that seek to promote children’s *pre-school intellectual enrichment* and *social skills* (e.g., Beelmann & Lösel, 2006; Farrington & Welsh, 2007; Welsh et al., 2012). Given the rationale behind the program evaluated in the current study, and its inclusion in the social skills training domain, it is now important to further understand what are the specific goals, targets, and focus of these programs.

### Social Skills Training Programs

Even though the concepts of social skills and social competence are usually used interchangeably, they reflect in fact different things. Social skills comprise more specific behavioral expressions inserted in a broader construct that is social competence. Usually, social competence is understood as a concept that integrates three levels of interactive competencies: cognitive competencies (e.g., social information processing), emotional competencies (e.g., age appropriate emotional development), and behavioral competencies (e.g., verbal and non-verbal communication skills). In turn, the term social skill is related to several dimensions of social competence, such as the ability to establish interpersonal interactions or to promote emotional support. Thus, the specific social, emotional, and cognitive skills are the target of these kinds of programs (Lösel & Bender, 2012; Webster-Stratton & Taylor, 2001).

Typically, social skills training programs comprise a limited number of sessions in which they seek to teach, among others, adequate (non-aggressive) methods of social perception, communication, emotion recognition, causal attribution, empathy, social perspective taking, alternative thinking, foreseeing consequences, anger management, problem solving, and conflict resolution (Lösel & Beelmann, 2007). The implementation of these programs is usually based on structured group sessions and activities, mainly resorting to more educational methodologies such as the discussion of hypothetical scenarios that represent conflict situations, modeling techniques, role-playing, positive reinforcement of appropriate behavior, games, audiovisual stimuli, and homework to promote the transfer of the skills acquired (Lösel & Bender, 2012).

Different prevention programs, entirely focused on the development of these skills, or incorporating them as a module in a broader framework, were proven to be effective in reducing children and youth antisocial behaviors and enhancing their social adjustment (e.g., *The Montreal Longitudinal and Experimental Study; Promoting Alternative Thinking Strategies; Stop Now and Plan; The Fast Track Program;* Augimeri et al., 2007, 2018; Conduct Problems Prevention Research Group, 1999a, 1999b, 2004; Greenberg et al., 1995, 2003; Tremblay & Craig, 1995; Tremblay et al., 1996). For example, the Beelmann and Lösel (2006) systematic review found that these programs had a positive effect on the reduction of children’s antisocial behaviors and the development of their social competence. Similar results were found in a more recent meta-analysis, in which the effect of social skills training programs was positive, despite being small, in the prevention of antisocial development (Beelmann & Lösel, 2021).

However, despite the overall positive results found in the literature, in order to enhance the benefits resulting from these programs, there are certain conditions that should be ensured, namely regarding the *group size* (the smaller the group, the greater the effectiveness of the program); *the program integrity* (the program effectiveness is enhanced when it is implemented by its creators or by a team of highly trained implementers); *the implementation strategies* (better results have been observed for interventions including cognitive-behavioral techniques); and *the participant’s level of risk/needs* (with greater effectiveness found for programs that intervene in children who present high levels of risk; Lösel & Beelmann, 2003; Lösel & Bender, 2012).

### The Need for Program Evaluation

In light of the exponential growth and the multiplicity of developmental preventive strategies, among which we found the social skills training programs, we have been witnessing the development of a movement that highlights the importance and the need for scientifically evaluating these programs, especially since the beginning of the 21st century. In fact, even though there is strong scientific evidence that vouches for the beneficial or promising nature of these prevention programs, suggesting that developmental prevention is worth it and well justified (Weisburd et al., 2017), there are also data revealing that not all of the preventive efforts are adequate or efficient in reducing antisocial behavior. In fact, some studies have even found iatrogenic effects of programs (e.g., Gottfredson et al., 2010; MacKenzie, 2013; McCord, 2003; Taheri & Welsh, 2016; Welsh & Rocque, 2014), therefore suggesting that the overwhelmingly positive message about developmental prevention programs should not hide a series of problems faced by these interventions (Lösel et al., 2013).

So, one can only ask: how can we know if a program is really working? It is in the face of this question that the development of program evaluations emerges as crucial. Gradually, it has been recognized that, while there is some success in identifying effective programs, there is still limited knowledge about the processes and mechanisms that lead a program to be successful. By acknowledging this, increased attention is drawn to the importance of a greater understanding of the active components of preventive strategies, in order to enable the development of solid empirical evidence regarding programs and practices, so that it is possible to identify those that effectively work, those that are more effective and those that cause harm rather than benefits (Eisner & Malti, 2012; Weisburd et al., 2017).

Therefore, evaluations carried out using experimental designs have special relevance, as they are considered the most robust scientific program evaluations. Following the canons of experimental criminology, these studies allow the establishment of a causal link between the intervention and the results, as well as calculating the effect size, when a causal relationship is found, by comparing the intervention and control groups (Currie, 2001; Farrington, 2006; Shadish et al., 2002). In fact, within the scope of program evaluation research, it is usually considered that randomized controlled trials are the “gold standard” of research designs. This is because randomization equalizes the groups on all measured and unmeasured variables, thereby maximizing internal validity, which is considered the most important factor in the context of program evaluation (Farrington, 2003b, 2013; Lab, 2014; Lipsey et al., 2006; Rossi et al., 2004).

It is important to note that these evaluative studies are critical for decision-making in terms, for example, of abandoning ineffective practices and promoting efficient interventions or in choosing among a multitude of alternative programs. Impact evaluations are particularly relevant when applied to new programs that seek to test an unproven but promising approach, such the one developed in the current study. Likewise, this type of evaluation is important when substantial changes are made to an existing program, as it virtually becomes a new intervention (Gertler et al., 2011; Rossi et al., 2004).

### The Current Study

It is based on this rationale, and acknowledging the importance of properly evaluating new intervention programs, that the current study was planned and developed. This is particularly important considering that there have been relatively few experimental evaluations of programs in Portugal, especially concerning interventions with elementary-school children. Using a randomized controlled trial and a multi-measure and multi-informant approach (for a review of factors supporting this approach, see Santos et al., 2020), the current study sought to conduct an impact evaluation of the early developmental prevention program ZARPAR—Educational Program for Cognitive and Behavioral Development. In accordance with this goal, and considering the skills and competences developed and the expected results of the program, the following hypotheses were tested: (1) in comparison with the control group, the intervention group presents lower levels of attention problems, impulsivity, externalizing behaviors, both aggressive and rule breaking, and social problems, 6 months after the intervention; (2) in comparison with the control group, the intervention group presents higher levels of attention focusing, inhibitory control, effortful control, pro-social behavior and empathy, 6 months after the intervention.

## Method

### Participants

The study was undertaken with a community sample. The participants were non-referred children, attending the second grade of elementary school, in Porto, Portugal. Regarding the pre-test evaluation (Time 1—“*T*1”), the sample consisted of 103 children, *n* = 37 in the intervention group (IG) and *n* = 66 in the control group (CG). The average age at the first measurement was 6.80 (*SD* = 0.471; 51.5% boys) in the CG (ranging between 6 and 8 years old) and 7.30 (*SD* = 4.63; 59.5% boys) in the IG (ranging between 7 and 8 years old). No significant differences between groups were found concerning children’s sex. However, the intervention and control groups were found to be significantly different regarding the participant’s average age (*p* < .001). Regarding the post-test evaluation (Time 2—“*T*2”), the number of participants in the intervention (*n* = 34; 58.8% boys) and control (*n* = 61; 49.2% boys) groups decreased to a total of *n* = 95. This was mainly due to the transfer of children to other schools that did not participate in the study, being therefore very difficult to reach.

**Figure 1.**
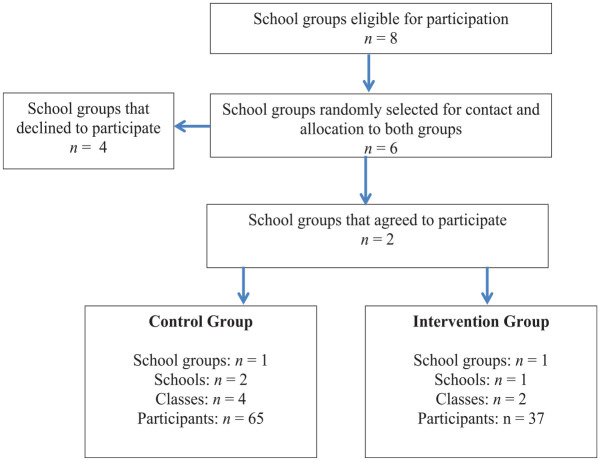
Sample selection procedures.

The participating children were selected from schools possessing particular features and thus receiving the classification of TEIP—school territories of priority intervention. These schools are situated in socio-economically disadvantaged areas, frequently marked by poverty, social exclusion, indiscipline, school truancy and dropout, and low school achievement. The selection process had several stages. First, a list of all the school groups^1^ in the municipality of Porto who were eligible for participation in this research was drawn. After that, three school groups were randomly selected for each condition, both intervention and control. After establishing contacts with these school groups, four declined to participate and two agreed to participate in the study. One was randomly assigned to each condition. The specific participating schools (within a given school group) and classes (of each school) were then jointly selected with the boards of each school group. The criteria underlying this selection process were concordant with the ones defined for the research project. Specifically, the schools were selected based on the grades they taught—only the schools within the school group teaching second grade levels were eligible. Furthermore, since the intervention program was administrated during curricular time, the board of each school group had to ensure the agreement of the teachers in charge of the classes eligible for participation in the current study, since it would directly affect their teaching plan. Thus, the classes and schools selected were those for which these requirements were met. Figure 1 illustrates the steps undertaken in this selection process.

In both times, the data was gathered using parent/legal guardian and classroom teacher ratings. It should be noted that, for the purposes of the current study, the person answering the questionnaire, either the parent or the legal guardian, is labeled the parent. In time 1, *n* = 65 respondents of the control group participated in the study (average age of 37.37; *SD* = 6.68), from which 75.4% were females and 93.8% were biological parents. A similar pattern was found for the intervention group (*n* = 36), in which the respondents (average age 36.82; *SD* = 9.56) were mainly female (70.6%) and biological parents (88.8%) of the participating children. No significant differences were found concerning respondents’ sex and age between control and intervention groups.

Regarding teachers, in time 1 a group of six individuals in the control and two in the intervention groups participated in the study. As for time 2, the number of participating teachers increased in the intervention group (*n* = 4), since a few children flunk the school year and are integrated in different classes than the ones assessed at the first measurement. As with the parents, most of the teachers in this study were female (*n* = 5 in *T*1; *n* = 6 in *T*2).

### Data Collection Procedures

The data was collected between September and December 2017 (*T*1) and during December 2018, specifically 6 months after the intervention (*T*2), which was implemented from January until June 2018. After the above mentioned sample selection procedures, the contact with the boards of each school group also aimed at ensuring the teachers’ agreement for their own participation in the study, and contacting parents for obtaining their informed consent and agreement to participate in the research, as well as the authorization for teachers to provide us with information about their children. Written informed consents were obtained from the parents of the participating children, as well as from teachers. It should be noted that these forms explicitly requested consent for participating in all phases of data gathering.

The questionnaires, details of the study and instructions were personally delivered and explained, to both parents and teachers, by the researchers. Parents and teachers completed paper and pencil self-report measures without any further guidance or the presence of the researchers. In each data-gathering phase, deadlines were defined for the respondents to deliver the questionnaires to the research team, taking into account the goals established for the current study and the time limits methodologically defined for it.

Regarding the data gathering, it is important to note that the current research project comprised both an impact and a process evaluation, considering the need for a greater understanding of the several elements that could explain the results. According to Tilley (2002), when combined with an impact evaluation, a process evaluation is a useful tool for providing information on how the intervention was implemented, whether or not it was developed within the necessary standards for success, how well the experimental group received the intervention, or what factors may have kept the program from achieving its goals and other similar concerns. Particularly, there are some typical factors that are considered in these evaluations, namely: program integrity, dosage, quality, adaptation, integration in the implementation context, responsiveness of participants and program reach (Durlak & DuPre, 2008; Lab, 2014). Naturally, all of these constitute specific social, physical and situational factors and conditions that might affect the likelihood of a prevention program to have an impact (Tilley, 2002). To this end, the process evaluation developed within the current study had three main goals: to analyze the integrity of the program, to understand the context in which the program was developed and to explore the challenges faced during the implementation. To gather the data, a multi-method (e.g., interviews, focus-group, and documental analysis) and multi-informant (e.g., participating children, teachers, and implementers) approach was adopted. It should be noted that, even though the analysis of the process evaluation conducted and its results is not the focus of the current article, it was considered important to state that it was developed, since the information obtained from it is highly important when interpreting the results found in the impact evaluation, as discussed later.

### Measures and Variables

A multi-informant, multi-measure assessment battery included teacher and parent ratings. Outcome measures represented the three program’s core domains: (a) behavioral problems, (b) executive functioning, and (c) social skills.

*Behavioral problems—*three variables were assessed within this domain: aggressive behavior, rule breaking behavior and the total externalizing behaviors score. Two instruments were used to assess these variables: The Child Behavior Checklist (CBCL/6-18), completed by parents, and the Teacher Report Form (TRF), completed by teachers (Achenbach & Rescorla, 2001). Both the CBCL and TRF are well-recognized standardized screening tools used to identify emotional, social, and behavioral problems and social competencies in children and adolescents. Both measures include two empirically derived broadband scales: the broadband internalizing scale, which is a measure of emotional problems, containing three syndrome scales: anxious/depressed, withdrawn/depressed, and somatic complaints; and the broadband externalizing scale that assesses behavioral problems and is composed of the rule breaking behavior and aggressive behavior syndrome scales (used in the current study for measuring behavioral problems).

Three other syndrome scales do not belong to any of the broadband scales mentioned, namely: social problems, thought problems, and attention problems (Achenbach & Rescorla, 2001). For each syndrome scale, participants were requested to report on the degree or frequency of each one of the behaviors assessed, using a scale varying from 0 = “*not true*,” 1 = “s*omewhat or sometimes true*,” to 2 = “*very true or often true.*” The standard rating period for the CBCL is the last 6 months, while for the TRF it is the last 2 months. Both instruments were translated and validated for the Portuguese population, revealing good psychometric properties (see Fonseca et al., 1994, 1995). In the current study Cronbach’s alpha reliability coefficients for the CBCL scales were as follows: aggressive behavior α = .86 (*T*1) and α = .86 (*T*2); rule breaking behavior α = .64 (*T*1) and α = .66 (*T*2); and for the broadband externalizing scale α = .88 (*T*1) and α = .88 (*T*2). For the TRF scales used, the Cronbach’s alpha reliability coefficients were: for aggressive behavior α = .95 (*T*1) and α = .95 (*T*2); for rule breaking behavior α = .79 (*T*1) and α = .77 (*T*2); and for the broadband externalizing scale α = .96 (*T*1) and α = .96 (*T*2).

*Social skills—*three dimensions were assessed in this domain, namely: pro-social behavior, social problems, and empathy. Pro-social behavior was assessed using the teacher and parent version of the Strengths and Difficulties Questionnaire (SDQ; Goodman, 1997; Goodman et al., 1998; translated and adapted into Portuguese by Fleitlich et al., 2005). This measure was conceived to assess child and adolescent (4–16 years old) behaviors and emotions and it comprises 25 items designed to evaluate emotional symptoms, peer problems, hyperactivity, conduct problems, and pro-social behavior. Considering the purposes of the current study, only the pro-social behavior (e.g., “*kind to younger children*”) scale was used. Responses for each item were coded on a 3-point Likert scale, ranging between 0 = *not true* and 3 = *certainly true* (Goodman 1997; Goodman et al., 1998). The Cronbach’s alpha reliability coefficients were α = .73 (*T*1) and α = .82 (*T*2) for parent report; and α = .95 (*T*1) and α = .88 for teacher report.

Empathy was assessed using the Griffith Empathy Measure (GEM; Dadds et al., 2008; Wied et al., 2010). The GEM was adapted from Bryant’s Index of Empathy for Children and Adolescents (Bryant, 1982) and consists of a 23-item self-report measure of empathy, in which parents are asked to provide a series of answers regarding their children’s behaviors and attitudes on a nine-point Likert scale ranging from −4 = “*strongly disagree*” to +4 = “*strongly agree.*” The GEM has been widely used and validated as a total score and subscales for cognitive and affective empathy (Dadds et al., 2008). In the current study, only the total empathy score was used. The Cronbach’s alpha reliability was α = .75, for both time 1 and time 2.

Regarding social problems, they were assessed using both CBCL and TRF (previously described) social problems syndrome scales, which were designed to assesse immature social behaviors, as well as interpersonal difficulties (e.g., “*poor peer relations*”; Achenbach & Rescorla, 2001). In the current study Cronbach’s alpha reliability coefficients for the social problems scale were α = .73 (*T*1) and α = .63 (*T*2) for the parent report; and α = .77 (*T*1) and α = .72 (*T*2) for the teacher report.

*Executive functioning—*five variables were measured within the executive functioning domain: attention focusing, inhibitory control, effortful control, impulsivity, and attention problems. All variables were assessed using the Temperament in Middle Childhood Questionnaire (TMCQ; Simonds, 2006; Simonds & Rothbart, 2004), the only exception being attention problems, which were evaluated using both the CBCL and TRF forms (previously described), yielding the following reliability scores: α = .88 (*T*1) and α = .85 (*T*2) for parent report; and α = .96 (*T*1) and α = .94 (*T*2) for teacher report. The TMCQ (Simonds, 2006; Simonds & Rothbart, 2004) is a caregiver self-report measure developed to assess temperament in children aged between 7 and 10 years old. It includes 157 items, based on which parents are asked to describe their children using a five-point Likert scale, ranging from 1 = “*Almost always untrue*” to 5 = “*Almost always true*,” with “*Does not apply*” as an additional option. From the 17 dimensions of temperament assessed by the included in this measure, 13 were adapted from the well-validated Children’s Behavior Questionnaire (CBQ; Rothbart et al., 2001).

In the current study we restricted the dimensions that were analyzed in order to meet the research purposes, thus including: *attention focusing* (i.e., the ability to maintain attentional focus in a given task and to shift the attention when needed); *inhibitory control* (i.e., the ability to plan and suppress an inappropriate response when told or when facing new or uncertain situations); *impulsivity* (i.e., speed of response initiation) and *effortful control*, which is a factor scale driven from the computation of weighted averages of different scales integrating TMCQ, namely: attention focusing, inhibitory control (previously presented), but also low intensity pleasure (i.e., pleasure or joy driven from activities that involve lower levels of intensity, frequency, complexity, novelty, or incongruence); perceptual sensitivity (i.e., the ability to detect slight, low intensity stimuli from the external environment), and activation control (i.e., the capacity to perform an action when there is a strong tendency to avoid it; Simonds, 2006; Simonds & Rothbart, 2004).

It should be noted that, in the current study, it was considered important to gather the teacher reports on the dimensions assessed by the TMCQ. So, after obtaining the authorization and guidelines from the instrument authors, and in order to include teachers as respondents, the authors adapted the parent form of the TMCQ to a teacher report version. It should be mentioned that the Cronbach’s alpha reliability coefficients were considered acceptable or good on all dimensions assessed, based either on the parent (attention focusing: α = .84 (*T*1); α = .86 (*T*2); inhibitory control: α = .62 (*T*1 and *T*2); impulsivity: α = .83 (*T*1) and α = .76 (*T*2); and effortful control: α = .84 (*T*1) and α = .86 (*T*2)); or teacher (attention focusing: α = .97 (*T*1); α = .94 (*T*2); inhibitory control: α = .76 (*T*1) and α = .77 (*T*2); impulsivity: α = .95 (*T*1) and α = .85 (*T*2); and effortful control: α = .94 (*T*1) and α = .89 (*T*2)).

### The Intervention Program: “ZARPAR—Educational PROGRAM for Cognitive and Behavioral Development”

The development of this program was based in the fundamental and applied scientific knowledge that, since the 1990’s, supports the rationale underlying the developmental prevention model, based on the key factors and processes underlying the development of antisocial, problematic and delinquent behaviors throughout life; and the design, implementation and evaluation of prevention programs that seek to reduce risk factors and to promote protective ones (e.g., Moffitt et al., 2011; Welsh & Farrington, 2012). Following this rationale, “ZARPAR” constitutes a pilot intervention that was developed as a universal, individual-level, social and cognitive skills training program, seeking to promote children’s behavioral adjustment, by enhancing pro-social behaviors and reducing disruptive ones. In order achieve this, and in line with the skills frequently developed within these programs, as described in the introduction section of this article, the program comprises six main modules, developed simultaneously and in different sessions throughout the program, being largely focused on the development of children’s: (i) executive functioning (attention, memory, inhibitory control, cognitive flexibility); (ii) planning skills (setting goals, making decisions, setting priorities in problem solving, anticipating consequences); (iii) strategies to deal with frustration, to control aggression, to solve interpersonal problems and to manage conflicts (e.g., choosing alternatives to aggression, negotiation); (iv) communication and interpersonal skills (e.g., listening, starting a conversation, introducing yourself, greeting, thanking, asking for help, apologizing); (v) empathy, taking a social perspective, recognizing and understanding others’ emotions, feelings and needs; and (vi) moral values (e.g., honesty, sharing, solidarity, justice and equity, respect for the needs, and rights of others).

The development of these dimensions is frequently associated with the improvement of children’s school performance, the reduction of inappropriate behaviors and the promotion of pro-social ones. These aspects are crucial throughout different developmental stages of a child’s life course, since they might exert an influence on the prevention of risky and anti-social behaviors in adolescence, on the promotion of healthy life styles, and on the acquisition of relevant learning within the process of social integration and interaction.

ZARPAR is suitable for implementation in different contexts, with the school setting being one of them. In the current study, the program, comprising 18 structured sessions, was implemented with elementary school children, attending the second grade, being delivered in the classroom, and integrated into the school curriculum as a “complementary offer.” The intervention group was divided into small groups of about 10 children and the program was delivered by four implementers (two for each group), in weekly group sessions, each lasting approximately 1 hour, over 6 months. It should be noted that all the implementers had adequate college training (i.e., in criminology), previous experiences working with children in social settings and had a broad knowledge of the program’s rationale, goals, structure, and activities provided by the program’s coordination team that promoted several training sessions before the beginning of the intervention.

Each session included a set of activities conceived to be carried out by and with the children. The activities were designed taking into account not only the participant’s developmental stage and set of skills, but also the specific goals set for each module. Like several other programs in the skills training domain (as previously described), ZARPAR privileges the use of pedagogic, dynamic, playful and interactive activities, resorting to techniques such as discussion of scenarios, modeling, role-playing, or reinforcement of positive behaviors. For example, session eight of the program is focused on the development of interpersonal conflict resolution (by accepting differences) and problem solving (emphasizing the importance of paying attention in this process) skills. In order to achieve these aims, different activities are planned, including a group discussion after the viewing of a film focused on interpersonal conflict resolution, discussion of a hypothetical scenario in which a character is discriminated against based on his appearance, and a game in which the children are required to stay focused and exercise attention in order to find each matching pair.

Finally, it should be mentioned that the control group were not exposed to any activity within the program, receiving the usual school curriculum activities during the “complementary offer” time, throughout the entire duration of the project.

### Data Analyses

Descriptive statistics and reliability analyses of the scales were used to assess the psychometric features of the sample. Scale scores were displayed to reflect actual distributions and were computed through the sum of all item scores on each scale, and then by dividing them by the total number of items in the specific scale. To explore the effect of the intervention program, independent-sample t-tests were used to analyze group differences both prior (analyzing the group equivalence) and after the intervention. A measure of effect size—Cohen’s *d—*was calculated, thus allowing the understanding of the actual size of the differences observed between groups in each time, and the calculation of scores comparing the pre-post change of intervention and control groups (intervention effect; Durlak, 2009). Additionally, paired samples *t*-tests were also used to compare the scores of the assessed dimensions. These comparisons make it possible to assess the change in scores in each group between pre and post-test. Once again, Cohen’s *d* was used in order to further explore the size of the observed changes. It should be noted that *p*-values for multiple tests were not corrected given that several studies state that effect sizes should be used, rather than *p*-values, in indicating the strength of relationships, since they depend crucially on sample size (e.g., Sullivan & Feinn, 2012; Trafimow & Marks, 2015; Wasserstein & Lazar, 2016). In line with this, no power analysis was carried, since it aims to specify the minimum sample size that is needed to achieve a particular *p*-value.

## Results

### Preliminary Analysis

To examine pre-test differences between the intervention and the control groups, independent-sample t-tests were conducted. Considering the parent report, results showed that for the majority of variables assessed there were no differences prior to the intervention between both groups, as displayed in Table 1. The only exception concerned social problems (*p* = .004; *d* = −0.72), regarding which there was a significant difference between intervention and control groups, with the former scoring higher and thus suggesting higher levels of social problems in T1 and a lack of equivalence between groups for this dimension.

**Table 1.** Intervention Effects in T2 According to Parent Report: Comparison Between Groups.

Parent report										
	CG	IG	Group difference (T1)		CG	IG	Group difference (T2)		Intervention effect	
	Pre *M* (*SD*)	Pre *M* (*SD*)			Post *M* (*SD*)	Post *M* (*SD*)			ES *T*2–*T*1	
			*d*	*p*			*D*	*p*	*d*	*p*
Behavioral problems										
Aggressive behavior	1.29 (0.24)	1.32 (0.30)	−0.08	.69	1.22 (0.23)	1.31 (0.25)	−0.41	.06	−0.34	.12
Rule breaking behavior	1.09 (0.09)	1.15 (0.18)	−0.46	.07	1.09 (0.11)	1.12 (0.16)	−0.29	.19	0.19	.44
Externalizing behaviors	2.38 (0.31)	2.46 (0.45)	−0.22	.27	2.30 (0.31)	2.44 (0.39)	−0.39	.07	−0.15	.48
Executive functioning										
Attention problems	1.46 (0.37)	1.61 (0.49)	−0.36	.09	1.38 (0.33)	1.53 (0.40)	−0.43	**.05**	0.008	.97
Attention focusing	3.13 (0.89)	3.20 (0.93)	−0.08	.71	3.15 (0.89)	3.06 (1.02)	0.10	.64	0.21	.33
Impulsivity	3.17 (0.66)	3.23 (0.66)	−0.08	.69	3.13 (0.59)	3.02 (0.67)	0.17	.44	0.24	.33
Inhibitory control	3.29 (0.69)	3.32 (0.46)	−0.05	.81	3.47 (0.56)	3.29 (0.59)	0.31	.15	0.30	.17
Effortful control	3.45 (0.47)	3.38 (0.40)	0.15	.48	3.51 (0.41)	3.35 (0.45)	0.39	.07	0.22	.32
Social competence										
Social problems	1.27 (0.21)	1.46 (0.34)	−0.72	**.004**	1.21 (0.20)	1.35 (0.21)	−0.72	**< .001**	0.17	.44
Pro social behavior	2.69 (0.35)	2.67 (0.41)	0.07	.76	2.72 (0.37)	2.71 (0.40)	0.01	.95	0.37	.08
Empathy	1.75 (0.84)	1.78 (0.86)	−0.04	.86	1.80 (0.87)	1.58 (0.78)	0.25	.25	0.37	.09

*Note*. CG = control group; IG = intervention group; *M* = mean; *SD* = standard deviation; *d* = Cohen’s *d* effect size measure; ES = effect size; *T*2 = time 2; *T*1 = time 1. Significant differences are indicated in bold.

Regarding teacher reports, as presented in Table 2, significant pre-test differences between groups were observed on the aggressive (*p* = .02; *d* = −0.59), rule breaking (*p* = .03; *d* = −0.54), and total externalizing (*p* = .02; *d* = −0.59) behaviors scores, as well as for attention (*p* = .03; *d* = −0.46) and social problems (*p* = .001; *d* = −0.79), once again with the intervention group scoring higher in all of these dimensions. Thus, despite the adoption of a randomization strategy for selecting the participating schools (that ideally would ensure the equivalence between experimental and control groups prior to the intervention), the data suggested that the randomization process conducted was not effective in creating equivalent groups prior to the intervention on some dimensions, particularly based on teacher reports, and, therefore, the subsequent data interpretation should be conducted carefully.

**Table 2.** Intervention Effects in T2 According to Teacher Report: Comparison Between Groups.

Teacher report										
	CG	IG	Group difference (T1)		CG	IG	Group difference (T2)		Intervention effect	
	Pre *M* (*SD*)	Pre *M* (*SD*)			Post *M* (*SD*)	Post *M* (*SD*)			ES *T*2–*T*1	
			*d*	*p*			*D*	*p*	*d*	*p*
Behavioral problems										
Aggressive behavior	1.15 (0.27)	1.35 (0.47)	−0.59	**.02**	1.28 (0.39)	1.34 (0.47)	−0.13	.54	0.23	.28
Rule breaking behavior	1.15 (0.19)	1.29 (0.36)	−0.54	**.03**	1.18 (0.24)	1.21 (0.30)	−0.13	.53	0.22	.31
Externalizing behaviors	2.29 (0.43)	2.64 (0.81)	−0.59	**.02**	2.47 (0.61)	2.55 (0.73)	−0.14	.52	0.24	.26
Executive functioning										
Attention problems	1.35 (0.41)	1.55 (0.51)	−0.46	**.03**	1.51 (0.36)	1.68 (0.48)	−0.41	.08	−0.004	.99
Attention focusing	3.08 (0.79)	2.98 (1.40)	0.09	.69	2.97 (0.86)	2.42 (1.04)	0.60	**.01**	0.53	**.03**
Impulsivity	2.77 (0.64)	.277 (1.15)	0.003	.99	3.43 (0.76)	3.03 (0.88)	0.50	**.02**	0.32	.14
Inhibitory control	3.98 (0.67)	4.11 (0.73)	−0.18	.37	3.91 (0.94)	3.71 (0.81)	0.22	.30	0.37	**.05**
Effortful control	3.44 (0.48)	3.42 (0.65)	0.04	.86	3.47 (0.76)	3.15 (0.61)	0.45	**.04**	0.45	**.04**
Social competence										
Social problems	1.13 (0.19)	1.31 (0.30)	−0.79	**.001**	1.23 (0.24)	1.33 (0.26)	−0.44	**.04**	0.18	.40
Pro social behavior	2.36 (0.49)	2.24 (0.66)	0.21	.34	2.38 (0.46)	2.33 (0.58)	0.09	.68	−0.04	.84

*Note*. CG = control group; IG = intervention group; *M* = mean; *SD* = standard deviation; *d* = Cohen’s *d* effect size measure; ES = effect size; *T*2 = time 2; *T*1 = time 1. Significant differences are indicated in bold.

### Post-Test Between-Group Differences

Table 1 presents the results of the independent sample *t*-tests conducted to analyze post-test between-group differences, according to the parent report. As displayed in the table, the data indicates significant differences between groups regarding the social (*p* < .001; *d* = −0.72) and attention (*p* = .05; *d* = −0.43) problems dimensions, with the intervention group scoring higher for both and thus suggesting worse results after the intervention. However, in both cases the scores decreased from pre-test to post-test, indicating no effect of the intervention. No other significant differences were found.

On the dimensions that showed statistically significant group differences after the program, the intervention effect ranged from *d* = 0.00 (*p* = .97) to *d* = 0.17 (*p* = .44), thus reinforcing the non-significant intervention effect.

Regarding the teacher report, the analysis of post-test between-group differences, displayed in Table 2, revealed significant differences concerning attention focusing (*p* = .01; *d* = 0.60) and effortful control (*p* = .04; *d* = 0.45), with the control group scoring higher for these dimensions and thus suggesting worse results in *T*2 for the intervention group. Furthermore, there were significant differences between groups regarding social problems (*p* = .04; *d* = −0.44), with the intervention group scoring higher and thus indicating worse results. However, as the intervention group was worse at the pre-test, it is more plausible to suggest that the program had no effect on social problems. The only exception concerned impulsivity (*p* = .02; *d* = 0.50) for which the intervention group was significantly lower, thus suggesting better outcomes after the intervention, in comparison to the control group.

Similarly to what was found for parents, on the dimensions that showed statistically significant group differences after the program, the intervention effect varied from *d* = 0.18 (*p* = .40) to *d* = 0.53 (*p* = .03) supporting the existence of small to medium intervention effects.

### Post-Test Within-Group Differences

It should be mentioned that besides comparing intervention and control groups in *T*1 and *T*2, the current analysis also sought to explore the post-test within-group differences, in order to further examine the changes observed in each group between pre-test and post-test. Table 3 displays the results obtained based on parent reports. Similarly to what was observed in the between-group comparisons, no differences were found for the majority of the dimensions assessed in both groups between T1 and T2. Specifically, regarding the control group the only significant difference was found for aggressive behaviors (*p* = .05; *d* = 0.26), indicating a decrease in these behaviors as suggested by the mean scores presented in Table 1.

**Table 3.** Intervention Effects in T2 According to Parent Report: Comparison Within Groups.

Parent report				
	Control group		Intervention Group	
	Difference		Difference	
	*d*	*p*	*d*	*p*
Behavioral problems				
Aggressive behavior	0.26	.05*	−0.06	.71
Rule breaking behavior	−0.07	.60	0.10	.55
Externalizing behaviors	0.18	.18	0.02	.91
Executive functioning				
Attention problems	0.17	.20	0.16	.37
Attention focusing	0.05	.70	0.23	.18
Impulsivity	0.04	.78	0.21	.23
Inhibitory control	−0.22	.11	0.08	.64
Effortful control	−0.008	.95	0.22	.21
Social competence				
Social problems	0.23	.09	34	.06.
Pro social behavior	−0.05	.69	−0.09	.60
Empathy	−0.03	.84	0.38	.04*

*Note. M* = mean; *SD* = standard deviation; *d* = Cohen’s *d* effect size measure.

* *p* < 05. Significant differences are indicated in bold.

As for the intervention group, the only significant difference found in the within-group analysis concerns empathy (*p* = .04; *d* = 0.38), signifying a decrease in the empathic behaviors and attitudes displayed by the participants in the intervention group between *T*1 and *T*2 as suggested by the mean scores presented in Table 1.

A similar analysis was also conducted based on teacher reports, as presented in Table 4. Regarding the control group, significant differences between *T*1 and *T*2 were found for aggressive behaviors (*p* = .004; *d* = −0.38), externalizing behaviors (*p* = .01; *d* = −0.33), attention problems (*p* = .002; *d* = −0.41), impulsivity (*p* < .001; *d* = −0.75), and social problems (*p* = .004; *d* = −0.39), suggesting worse results in these dimensions in *T*2.

**Table 4.** Intervention Effects in *T*2 According to Teacher Report: Comparison Within Groups.

Teacher report				
	Control group		Intervention group	
	Difference		Difference	
	*d*	*p*	*d*	*p*
Behavioral problems				
Aggressive behavior	−0.38	.004**	−0.16	.35
Rule breaking behavior	−0.20	.12	0.04	.84
Externalizing behaviors	−0.33	.01*	−0.08	.64
Executive functioning				
Attention problems	−0.41	.002**	−0.48	.008*
Attention focusing	0.19	.14	0.56	.003**
Impulsivity	−0.75	<.001**	−0.31	.07
Inhibitory control	0.06	.65	0.63	<.001**
Effortful control	0.003	.98	0.51	.005*
Social competence				
Social problems	−0.39	.004**	−0.20	.26
Pro social behavior	0.00	1.00	−0.04	.82

*Note. M* = mean; *SD* = standard deviation; *d* = Cohen’s *d* effect size measure.

** *p* < .01. *p < .05. Significant differences are indicated in bold.

Concerning the intervention group, as presented in Table 4, significant differences were only observed for the executive functioning domain, specifically regarding attention problems (*p* = .008; *d* = −0.48), attention focusing (*p* = .003; *d* = 0.56), inhibitory control (*p* < .001; *d* = 0.63), and effortful control (*p* = .005; *d* = 0.51), indicating a decrease in these behaviors as suggested by the mean scores presented in Table 2 and, thus, worse results in *T*2.

## Discussion

As previously discussed, over the last decades a plethora of early developmental prevention programs and strategies have been developed and implemented aiming at enhancing the social, emotional, cognitive, and behavioral adjustment of children and youth (Farrington et al., 2016, 2017). Many of them have been considered effective or promising, but, it is also known that some initiatives are yet lacking evaluation and that some of these interventions do not achieve their intended goals, with some existing programs associated with iatrogenic outcomes (McCord, 2003; Rhule, 2005). This enhances the need for comprehensive, systematic, and rigorous evaluations (Royse et al., 2010; Weisburd et al., 2017; Welsh & Farrington, 2012).

Therefore, using a multi-measure and multi-informant approach, the current study sought to conduct an impact evaluation for the pilot early developmental prevention program ZARPAR. Specifically, it was hypothesized that, after the intervention, the treatment group would present better outcomes regarding the program’s central components. Particularly it was expected that, after the program, the intervention group would present lower levels of attention problems, impulsivity, externalizing behaviors, both aggressive and rule breaking, and social problems; and higher levels of attention focusing, inhibitory control, effortful control, pro-social behavior, and empathy, in comparison with the control group. However, contrary to what was expected, the data largely suggested no effect of the intervention or, for some dimensions, even the existence of negative outcomes. The only exception was teacher-rated impulsivity, for which the intervention group presented better outcomes after the intervention, as hypothesized.

These results are in line with some findings from previous studies. Perhaps the most relevant example of a program that had iatrogenic effects is the classical *Cambridge-Somerville Youth Study* (McCord, 2003). Contrary to expectations, the results of the evaluation, conducted using an experimental design, showed that participants in the experimental group exhibited more unfavorable results compared to those in the control group. Experimental children had, for example, higher levels of criminal convictions, a higher mortality rate before age 35 years, and more alcohol consumption problems (for a complete review of the effects see McCord, 1992, 2003).

Also, when evaluating interventions aimed at preventing youth antisocial behaviors, Dishion and Andrews (1995) found that, according to maternal reports, the results suggested no effect of the intervention program, since no differences were found between experimental and control groups regarding externalizing and antisocial behaviors. Furthermore, considering the teachers’ reports, the experimental group presented higher levels of externalizing behaviors, compared to the control condition, thus suggesting an undesirable effect of the intervention.

Another example is the quasi-experimental study developed by Weisman et al. (2002) that sought to evaluate several after-school programs, which revealed that the participants in this type of program had higher levels of delinquency, rebelliousness, substance use and association with deviant peers, compared to the control groups. In line with this finding, a more recent systematic review and meta-analysis showed that evaluated after-school programs had small or non-significant effects in preventing delinquent behavior (about 3%; Taheri & Welsh, 2016).

Lipsey’s (1992) meta-analysis also found that around 29% of interventions that were focused on reducing behavioral problems presented by young people showed adverse outcomes, which were more frequent when the interventions were implemented within the peer group (e.g., group counseling, residential intervention or interventions developed in the school context; Farrington & Welsh, 2003).

In a more recent study, Welsh and Rocque (2014) conducted a review of systematic reviews on individual developmental prevention programs and concluded that, although there were some programs that had adverse outcomes, the vast majority of them were related to the ineffectiveness of the intervention, that is, having no effect on the expected results.

Thus, it is easy to understand that the effectiveness of developmental prevention programs, although widely disseminated, is not a certainty and, therefore, the discussion of what might have prevented some programs from achieving their goals is necessary. Previous studies have provided a wide set of elements associated with null or negative program outcomes (e.g., the implementation context, the type of intervention strategies, the participant characteristics, the implementation process; Dishion et al., 1999; Gottfredson et al., 2010; Rhule, 2005; Weisburd et al., 2017; D. Wilson et al., 2008), many of which have emerged in the process evaluation developed within this study and could contribute to the understanding of these effects. For example, the observed results might be related to the specific context in which the program was developed (Dariotis et al., 2008; Payne & Eckert, 2010). As previously mentioned, ZARPAR was implemented in a school setting. This is a space that, despite its advantages, is frequently associated with limitations and difficulties regarding the implementation of such initiatives, thus affecting their success. Many of these problems emerged in the data resulting from the process evaluation conducted within the current study, for example: the concern with the reduction in children’s teaching time, deficiencies in the communication process between program staff and school agents, the availability of suitable spaces for conducting the sessions or the last minute schedule changes that are not articulated to the implementers (Dariotis et al., 2008; Farrington & Welsh, 2003; Jaycox et al., 2006; Payne & Eckert, 2010). In addition, there are other aspects of the specific context in which the program was developed that should be considered, namely the potential influence that some features of the schools sampled might have exerted in the results observed. For example, results from different studies revealed that the quality of the school climate (i.e., the quality of teaching and learning, the interpersonal relationships, the school organization and the structural characteristics of the school environment; Cohen et al., 2009; Kim, 2016; Wang & Degol, 2016) might influence the effects of school-based prevention programs, suggesting that a positive school climate could enhance the effectiveness of such interventions (e.g., Low & Van Ryzin, 2014; McCormick et al., 2015; Wang & Dishion, 2012). Therefore, even though the influence of such factors was not explored in the current study, thus limiting the possibility of establishing these type of connections, their importance should not be overlooked since the characteristics of the participating schools might, in fact, have contributed to the results observed.

Another element that should be considered as a contributing factor to the observed results is the group intervention strategy. In fact, even though the development of group programs is not always pointed out as a negative factor, and a plethora of research studies present successful group interventions (Rhule, 2005), this could be a potential explanatory factor, mainly due to the its association with the dynamics and difficulties inherent in group activities (e.g., the negative influence of the peer group, as found in the current study). This explanation is consistent with previous studies (e.g., Cécile & Born, 2009; Gottfredson, 2010). For example, a key factor in the undesirable effects of the Cambridge-Somerville Youth Study (McCord, 2003) was putting together antisocial boys. Also Welsh and Rocque (2014) found that three-quarters of the interventions presenting negative effects were developed in group contexts (in comparison with individualized studies).

Additionally, specific program implementation features, particularly regarding integrity, quality, reach, and dosage, should be considered as potential contributing factors for the lack of desirable results observed (e.g., Durlak & DuPre, 2008; Gottfredson et al., 2007; Lab, 2014; Patton, 2012a, 2012b; Small & Huser, 2016; Zane et al., 2016). The process evaluation conducted revealed that there were differences between the initial program plan and what was actually delivered, particularly concerning the competencies developed, the activities conducted, and the number of sessions implemented or actually attended by the children, thus suggesting that the lack of results may have been due to some extent to the implementation problems of some components of the program.

These results are consistent with previous studies. For example, Durlak and DuPre (2008) revealed that programs achieved better outcomes when they were adequately implemented, without deviations from the original plan. Also, S. Wilson et al. (2003) found that program integrity constituted the most important program feature in explaining the observed results. In the same vein, Duwe and Clark (2015) concluded that intervention programs were more effective when they upheld high standards of fidelity, that is, when they were implemented with high integrity and were developed as planned. On the other hand, whenever the program implementation process presented deviations from the original planning, for example, when certain program contents were not covered, when the duration of the program or the number of sessions was shortened or when the number of participants was higher than recommended, the desirable program effects were diminished.

Nevertheless, it should be mentioned that, despite the above discussed features, in the current study, the data resulting from the process evaluation revealed that the differences between the original program plan and the implementation were mainly related to the need to adapt the program activities and components to the participant’s needs and capabilities. These adaptations were introduced as a way of supplanting the participant’s difficulties in understanding and developing the activities and of enhancing their responsiveness, and should be considered while interpreting the data.

The aspects discussed above inevitably lead to a crucial thought: perhaps the null or negative effects of the program are not directly related to its nature and rationale, since it is similar to many interventions found to be effective or promising. Instead, consistent with previous studies, it might be argued that the unfavorable results were mostly related to certain implementation features and to the setting in which the program was developed. Furthermore, previous longitudinal studies have shown that the effects resulting from these kind of interventions might not be detected in short-term evaluations, as the one developed in the current study, thus suggesting that the observed results might change over time and that long-term evaluations are needed to better understand and assess the effect of a given intervention in achieving its proposed outcomes (Farrington, 2006; Lösel et al., 2013).

### Limitations

Despite the strengths of the current study, it is not immune to some limitations, especially concerning the small sample size. In fact, previous studies have suggested that larger samples allow the construction of more precise estimates, thus contributing to the development of more reliable conclusions about the relationships established between the program and the observed results. Often, small samples, such as those under study, cannot detect the existence of associations between the explanatory and outcome variables, due to their reduced statistical power (Farrington, 2003a; Lösel et al., 2013; Murray et al., 2009; Piquero et al., 2016). Therefore, future researches should ensure the inclusion of a greater number of participants in each condition, thus enhancing the statistical power and validity of the conclusions obtained. In line with this limitation, it should also be noted that only one school group was assigned to each condition. As previously discussed, the specific characteristics of a given school might exert an influence on the effectiveness of school-based prevention programs, such as the one evaluated in the current study. Thus, even though the exploratory nature of this study should be emphasized, it could be considered that the number of schools sampled might not have been sufficient to eliminate the influence of such differences on the results observed. Future studies should consider randomly sampling a larger number of schools to each condition, as well as controlling for the influence of specific school features, such as school climate.

Another limitation concerns the format of the adopted measures. In order to understand the intervention effect, the questionnaires were conceived with the aim of measuring the greatest possible number of program dimensions, considering its goals and expected results, which, in turn led to the inclusion of a large number of scales and subsequently to very long questionnaires. This might have impacted the quality of the reports provided, particularly regarding teachers, who had to evaluate entire classes, leading to more general and globalizing reports rather than individualized evaluations. Even though considered acceptable, it should also be noted that some subscales used in the current study, namely rule breaking behaviors, presented somewhat low reliability scores, thus reinforcing the need to interpret the results with caution. In future researches, this should be taken into account and equally reliable but shorter measures should be adopted.

The variation among respondents between pre-test and post-test, particularly regarding teachers, could also be perceived as a limitation, since it could increase the subjectivity inserted into the reports due to the influence of respondents’ individual traits, such as education, sex, age, or professional experience (Bodin et al., 2016; Lane et al., 2005). Therefore, future research should consider the influence of these variables, knowing that individual characteristics could be another potentially valuable for understanding the observed results.

Finally, one should consider the reduced accompanying of the control group’s activities and routines as a limitation of the current study. Besides the pre/post test data gathering phases, no follow-up was performed and no information was gathered about this group. Thus it is possible that aspects such as the deviation from the regular schedule, more attention, participation in other programs or peer interactions might have influenced the results observed. If so, factors such as these could contribute to the drawing of conclusions that refer to the null effect of the intervention, when this may not be the case. Future studies, should consider adopting more complex study designs (e.g., including not only experimental and control, but also observational groups; e.g., Tremblay et al., 2003; Welsh et al., 2012) in order to overcome such limitation.

Despite these limitations, mainly methodological, the fact that this was a pilot study and that the results are of a preliminary nature, mostly because of the constraints imposed by the small sample size and the difficulties following the randomization process, this is, to the best of our knowledge a pioneering evaluation study, using an experimental design, based on a sample of Portuguese elementary-school children. Furthermore, it seeks to highlight the need for rigorous evaluations, considering that, increasingly, we have watched the growth of multiple intervention programs based on the common-sense and wrong notion that “if it doesn't do good, it doesn't hurt either” (Welsh & Rocque, 2014). In fact, the current study emphasizes that not all interventions achieve their proposed goals and it has been an attempt to provide an extensive reflection of what might contribute to this. Ultimately, not only would this seek to help improve the program currently being evaluated, but it would also provide guidelines for other programs and evaluation plans being conceived, both at national and international levels, thus contributing to enhancing the methodological quality of these projects, as well as the success of future interventions.

## Notes

1. As per definition in the Law-decree no. 75/2008 of 22-04-2008, in its articles 1 and 2 “the school group is an organizational unit, with its own administration and management structures, consisting of pre-school education establishments and schools of one or more levels and cycles of education,” whose constitution follows the following criteria: “ (a) the development of integrated school pathways; (b) the curricular articulation between educational levels and study cycles; (c) geographical proximity; (d) the need to organize the network of pre-school, elementary and secondary education.”
